# Integrative transcriptome and WGCNA analysis reveal key genes mainly in response to *Alternaria alternata* in *Populus simonii × P. nigra*

**DOI:** 10.3389/fpls.2025.1540718

**Published:** 2025-02-17

**Authors:** Siyuan Liu, Lijuan Dai, Guanzheng Qu, Xinming Lu, Hong Pan, Xiaoyu Fu, Airong Dong, Libin Yang

**Affiliations:** ^1^ Key Laboratory of Biodiversity, Institute of Natural Resources and Ecology, Heilongjiang Academy of Sciences, Harbin, China; ^2^ State Key Laboratory of Tree Genetics and Breeding, Northeast Forestry University, Harbin, China; ^3^ College of Forestry, Northeast Forestry University, Harbin, China

**Keywords:** transcriptomic sequencing, *Populus simonii* × *Populus nigra*, WGCNA, key genes, *Alternaria alternata*

## Abstract

In order to explore the molecular mechanisms of *Populus simonii × P. nigra* response to stress and screen for genes conferring resistance to *Alternaria alternata*, we carried out measurements of physiological and biochemical indices and transcriptomic sequence analysis of leaves of *Populus simonii × P. nigra* inoculated with *A. alternata*. The results showed that the variation trends of multiple hormone contents and enzyme activities were broadly similar at different time points, with H_2_O_2_, SA, JA, PPO, SOD, PAL and POD showing a trend of increasing and then decrease after inoculation with the pathogen. The contents of H_2_O_2_ peaked on the second day and subsequently declined. The contents of SA and JA, as well as the enzymatic activities of SOD, PAL, and POD, reached their maxima on the third day before exhibiting a downward tendency. In contrast, the activity of PPO peaked on the fourth day. Whereas ABA content continued to increase until the fifth day and CAT content decreased and then increased. We subsequently identified 14,997 differentially expressed genes (DEGs) among the transcriptomic sequences(|log2FoldChange| > 1 and FDR value < 0.05), with genes encoding members of the ERF, MYB, bZIP, and WRKY transcription factor families being differentially expressed. Gene modules that were significantly associated with the ABA, PAL, JA, and SOD activity were identified using weighted gene co-expression network analysis (WGCNA). Gene Ontology (GO) and Kyoto Encyclopedia of Genes and Genomes (KEGG) enrichment analysis showed that these genes were mainly related to biological stress, signal transduction, cell wall, and photosynthesis. Within these modules, we also identified hub genes in the regulatory network, including GLK1/2 transcriptional activators, 14-3-3 proteins, cytosine 5 methyltransferases, and a variety of proteins associated with photosynthesis and respiration. This study showed that these hub genes, which play a pivotal role in the co-expression network, which may indicate a potential role in defense process of *Populus simonii × P. nigra* against *A. alternata*. Additionally, we analyzed the gene expression regulation and defense mechanisms of *Populus simonii × P. nigra* adversity stress, providing new insights into how plants respond to biological stress.

## 1 Introduction

Throughout their life cycle of growth and development, plants are constantly under stress from various pathogens in the environment. To fend off these pathogens, plants have developed a complex defense system (Jones and Dangl, 2006). The infection stage refers to a series of continuous processes in which pathogenic bacteria break through the physical and chemical defenses of plants through various means, successfully enter plant tissues, and colonize, grow, and reproduce within them. When in infection stage, plants initiate an immune response triggered by the recognition of pathogen-associated molecular patterns (PAMPs), which subsequently induces the transcription of related genes and the accumulation of secondary metabolites to help resist pathogen infection (Chakraborty and Newton, 2011; Sattler and Funnell-Harris, 2013; Bilir et al., 2019). In addition, some signaling molecules such as salicylic acid (SA), jasmonic acid (JA), and ethylene (ET) are also activated in the pattern-triggered immune (PTI) response as signals to trigger and regulate a series of defense responses. Some enzymes, such as peroxidase (POD) and superoxide dismutase (SOD), by controlling the redox condition of the plant cell (Umezawa et al., 2010; Robert-Seilaniantz et al., 2011; Truman and Glazebrook, 2012). Meanwhile, pathogens secrete effector proteins to counteract the immune PTI of PAMPs in plants, but these effector proteins can be recognized by the plant’s resistance (R) proteins, triggering effector-triggered immunity (ETI) (Tsuda and Katagiri, 2010; Cui et al., 2015). ETI can induce accumulation of SA, transcription of defense genes, and hypersensitivity reactions (HR). Transcription factors also play important roles in PTI and ETI, including basal expression of resistance components, direct transcription factor activity of receptor proteins, and activation downstream of receptor initiation (Wang et al., 2017b; Meraj et al., 2020). Overall, plant defense mechanisms are very complex and have different response strategies for different pathogens. Therefore, in-depth research into the response mechanisms of plants to different pathogens will provide a theoretical basis for improving plant disease resistance and screening for disease resistance genes.

In recent years, RNA-seq analysis has been widely used to identify differences in gene expression patterns in organisms and to reveal the functions of associated genes and proteins. Researchers have begun to focus on the analysis of pathogen response transcriptomes in many plants and crops. The genome of European ash trees has been sequenced using RNA-seq analysis technology, revealing associations with the degree of fungal infection caused by *Hymenoscyphus fraxineus* and the content of cycloartenoid glycosides (Sollars et al., 2017); By combining RNA-seq and biochemical data, researchers found that *Picea abies* triggers complex and dynamic regulatory changes in the hypersensitive response (HR) and phenolic pathways during infection by *Chrysomyxa rhododendri* (Trujillo-Moya et al., 2020). Su. et al. used transcriptome sequencing technology to elucidate the temporal expression patterns of genes in infected populations with *Fusarium* spp., revealing gene expression of poplar tree roots under different response patterns of the converting and converting enzyme inhibitor families in carbohydrate metabolism (Su et al., 2021). Currently, research on the interaction between poplar and pathogens mainly focuses on the study of poplar-rust (Petre et al., 2012; Li et al., 2016) and poplar-canker (Xing et al., 2020; Xie et al., 2023), while little is known about the defense mechanisms of poplar against other pathogens.

*Alternaria* spp. is a global genus of fungi, it usually manifests as necrotrophic when infected, members of which are commonly used as plant pathogens to infest a wide range of plants, including cereal crops, vegetables, and fruits, resulting in necrosis and defoliation of leaves, rotting of fruits and new shoots, shortening of the growth cycle, and postharvest rot (Ji et al., 2006; Garganese et al., 2016). At present, the control of *Alternaria* spp. is still mainly based on chemical control, but long-term use of fungicides can lead to problems such as resistance to microorganisms and environmental pollution (Yang et al., 2019; Srivastava et al., 2020). Therefore, a deeper understanding of plant defense mechanisms against *Alternaria* spp. through RNA-seq technology is expected to help design safer control strategies and contribute to the breeding of disease-resistant varieties. Researchers are currently investigating plant defense mechanisms against *Alternaria* spp. in a variety of plants. For example, when chrysanthemums were infected by the *Alternaria* spp (Zhao et al., 2020), the resistance (R) protein, ROS, calcium ions, mitogen activated protein kinase (MAPK), and JA signaling pathways were significantly increased. In addition, ET-/H_2_O_2_-mediated programmed cell death (PCD) and detoxification processes play a crucial role in the interaction between pear and *A. alternata* (Wang et al., 2017a); sequencing has shown that 28 candidate resistance genes with conserved leucine-rich repeat sequence (LRR) structural domains may contribute to the resistance of sand pear to *A. alternata* (Yang et al., 2015). Ethylene is likewise an important factor in citrus resistance to the development of *Alternaria* disease (Ortuño et al., 2008). These studies suggest that there are complex and diverse interactions between hosts and *Alternaria* spp. Poplar is a rapidly growing and highly productive tree species that is crucial for the ecological and socio-economic well-being of the world (Dickmann and Kuzovkina, 2014). Poplar leaf blight caused by *A. alternata* has become a common disease in China, with serious economic impacts. *Populus simonii × P. nigra*, as a hybrid species, is susceptible to *A. alternata* infestation leading to leaf blight disease. However, there are fewer studies on the molecular regulation of the infestation response of poplar to *A. alternata*, and the mechanism has not been clarified.

In this study, the physiological and biochemical indicators of different periods of pathogen infestation were first measured to understand the response of *Populus simonii × P. nigra* to *A. alternata* infestation. Subsequently, the expression of related genes was analyzed using the RNA-Seq technique. Candidate genes related to *A. alternata* resistance were further screened in combination with WGCNA. These results provide new insights into the molecular mechanisms of *Populus simonii × P. nigra* resistance to *A. alternata*, as well as candidate genes and theoretical foundations for the cultivation of long-lasting and broad-spectrum resistant poplar varieties in the future.

## 2 Materials and methods

### 2.1 Plant materials, pathogen culture, and inoculation methods

Inoculated plants were two-year-old asexual *Populus simonii × P. nigra* grown in the breeding base of the State Key Laboratory of Tree Genetics and Breeding, Northeast Forestry University (126.6342E, 45.7203N). Leaves from the second to fourth internode of *Populus simonii × P. nigra* plants with no significant difference in growth were selected, collected, and placed in a sealed bag, and kept on ice. The pathogenic bacterial cake *A. alternata* was incubated on PDA medium protected from light for 10 days until spore production (**Supplementary Figure S1**). The spore concentration was determined to be 1×10^8^ prior to inoculation. Holes were punched at the edge of the colonies using a 6 mm diameter punch in a clean bench, and the cakes were inoculated into healthy leaves using stab wound inoculation, with the leaves inoculated with the blank medium as the control (0 d), and the leaves inoculated with the pathogen cakes as the experimental group. The treated samples and controls were snap-frozen in liquid nitrogen on days 2, 3, 4, and 5 after pathogen inoculation and stored at -80°C for subsequent physiological and biochemical measurements (**Supplementary Figures S2A, B**), RNA extraction, and transcriptome sequencing. Three biological replicates were set up for each treatment.

### 2.2 Measurement of physiological and biochemical indicators

The contents of ABA, SA, JA, and H_2_O_2_, as well as the activities of the enzymes peroxidase (POD), polyphenol oxidase (PPO), superoxide dismutase (SOD), catalase (CAT), and phenylalanine ammonia-lyase (PAL) were measured in the plants of *Populus simonii × P. nigra* at different time periods (0 d, 2 d, 3 d, 4 d, 5 d). Using leaves that have not been inoculated with pathogen as CK (0d). Phytohormone content was determined using an enzyme-linked immunosorbent assay kit (ELISA kit, Quanzhou Ruixin Biotechnology, China) according to the manufacturer’s protocol. The activities of POD, SOD, PPO, PAL, and CAT were determined using the assay kits (Quanzhou Ruixin Biotechnology, China).The activities of PAL, SOD, POD as well as CAT were measured based on the methods of Liu (Liu et al., 2005). The PPO activity was measured according to the method of Hammerschmidt (Cavalcante et al., 2020). The H_2_O_2_ content was measured according to the method of titanium hydroxide complex method (Meng Ma et al., 2022). Three biological replicates set up for each assay.

### 2.3 RNA extraction, library construction, and sequencing

Total RNA from plant leaves was isolated using the Qiagen RNeasy Plant Mini Kit. DNA was digested using an RNase-Free DNase Set (Qiagen, Hilden, Germany) during the process of RNA extraction. The quality of RNA was checked by agarose gel electrophoresis to ensure that clear bands were visible. An Agilent 2100 Bioanalyzer was used to detect the integrity and quantity of RNA. The sampled *Populus simonii × P. nigra* leaves were sent with dry ice to Personalbio company (Shanghai, China) for high-throughput Illumina sequencing. The cDNA library was constructed and subsequently sequenced on the Illumina platform.

### 2.4 Analysis and functional annotation of differentially expressed genes

After removing splice-containing, low-quality reads and further filtering the sequencing data, the filtered reads were compared to the reference genome using the upgraded version of TopHat2, HISAT2 software (Kim et al., 2015; Pertea et al., 2015). In order to determine the response of *Populus simonii × P. nigra* to *A. alternata* infestation at different time points after inoculation, the expression level of each transcript was calculated based on FPKM (fragments per kilobase of exon per million mapped reads) using the software package Cufflink (Li and Dewey, 2011). Gene expression was analyzed differentially using DESeq. The conditions for screening differentially expressed genes (DEGs) were: expression difference multiplicity |log_2_FoldChange| > 1 and significance FDR-value < 0.05. PCA analysis was carried out using the Ropls package in R. GO enrichment analysis using topGO and KEGG enrichment analysis using clusterProfiler were used to identify GO terms and KEGG pathways that were significantly enriched for differential genes compared to the whole genome background (*Populus simonii × P. nigra* genome, unpublished).

### 2.5 qRT-PCR validation of gene expression

To validate the DEGs detected by RNA-seq sequencing, nine differentially expressed genes were randomly selected from 14,997 DEGs obtained from RNA-Seq for qRT-PCR analysis. And primers were designed using Primer Premier 6 and synthesized by Shanghai Bioengineering Biologicals, and the specific information of the primers is shown in **Supplementary Table 1**. qRT-PCR was performed on qTOWER3G (Jena Analytical Instruments GmbH, Germany) using a TB SYBR^®^ Premix Ex Taq™ II (Tli RNaseH Plus) kit (Takara RR820A). Three biological replicates per sample and three technical replicates per biological replicate were used with the *Populus simonii* × *P. nigra*. actin as the internal reference gene. Relative gene expression levels were measured using the 2^-ΔΔCt^ method (Schmittgen and Livak, 2008).

### 2.6 Weighted gene co-expression network analysis

By leveraging physiological and biochemical indicators along with transcriptome data, we will analyze trait-module relationships employing WGCNA technology. Gene expression levels were obtained by RNA-seq, and the R package WGCNA software was used to perform weighted association network analysis, construction of gene co-expression networks, calculation of topological properties of expression networks, data simulation, and visualization of data transformations (Langfelder and Horvath, 2008), and then Cytoscape (v3.8.2) was used to visualize gene network maps from this module. The minimum number of genes in a module was set to 30, and the merging threshold of similar modules was 0.8.

### 2.7 Statistical analysis

Data were statistically analyzed using SPSS 21 (Chicago, IL, USA). Significance was analyzed using t-test, with p-value <0.05 as the level of significance. Data were expressed as mean value ± standard error (SE) of three independent biological samples.

## 3 Results

### 3.1 Measurement of physiological and biochemical indicators of leaves after *A. alternata* infestation

**Figure 1:**
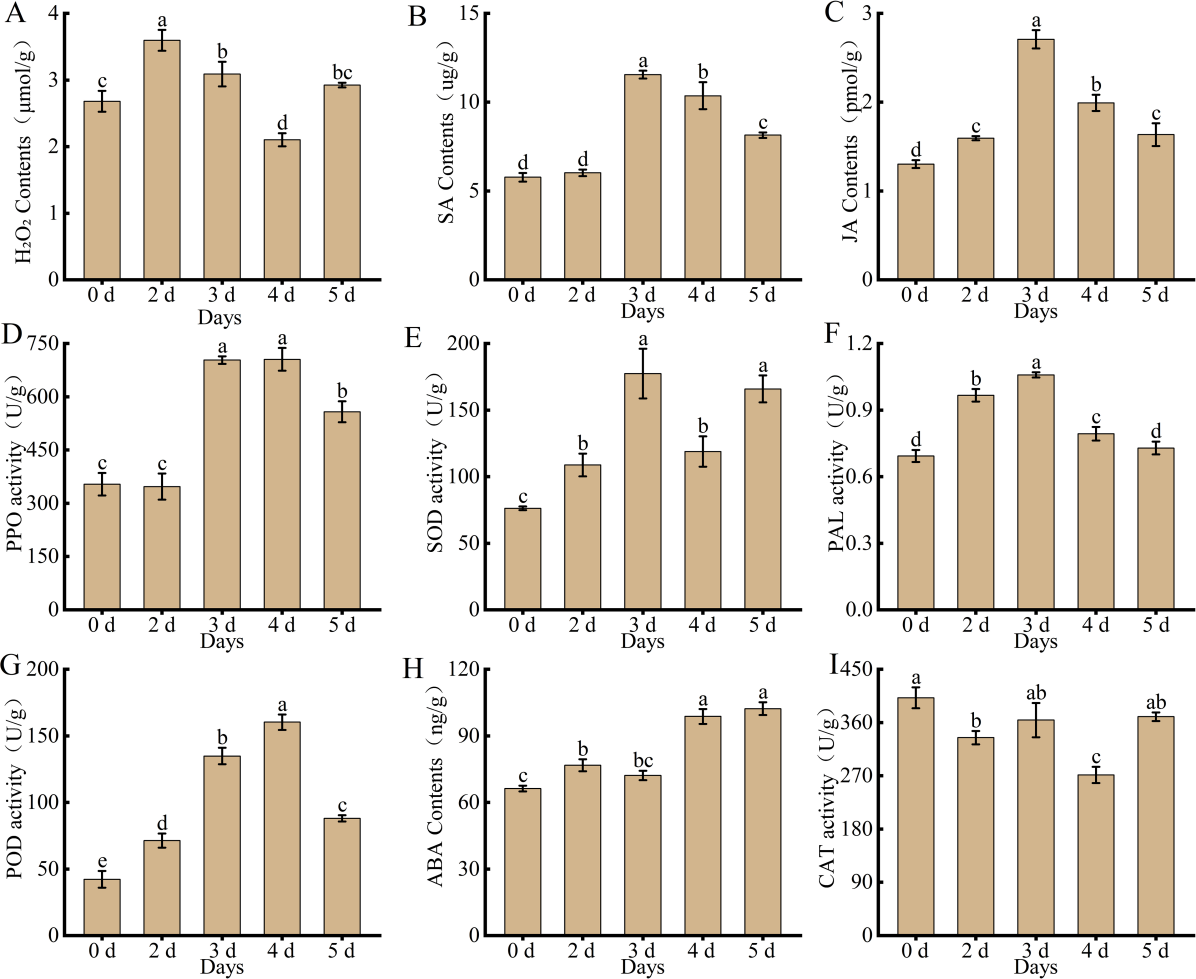
Changes in physiological and biochemical indexes of *Populus simonii × P. nigra* after inoculation with *A. alternata* **(A)** H_2_O_2_. **(B)** SA. **(C)** JA. **(D)** PPO. **(E)** SOD. **(F)** PAL. **(G)** POD. **(H)** ABA. **(I)** CAT. Different lowercase letters indicate significant differences (P < 0.05). The bars represent the means ± SEs from three independent experimental replicates.

Physiological and biochemical indices were measured to determine the status of hormones and ROS in the leaves of *Populus simonii × P. nigra* in response to *A. alternata*. The results showed (**Figure 1**) that the variation trends of multiple hormone contents and enzyme activities were broadly similar at different time points, with H_2_O_2_, SA, JA, PPO, SOD, PAL, and POD all showing a trend to increase and then decrease after inoculation with the pathogen. The contents of H_2_O_2_ peaked on the second day and subsequently declined. The contents of SA and JA, as well as the enzymatic activities of SOD, PAL and POD, reached their maxima on the third day before exhibiting a downward tendency. In contrast, the activity of PPO peaked on the fourth day. Whereas ABA content continued to increase until the fifth day and CAT content decreased and then increased. This suggests that these hormones and enzymes play an important role in the disease resistance of *Populus simonii × P. nigra* against *A. alternata*.

### 3.2 Sequencing quality analysis of transcriptome data

The transcriptome sequencing data of individual samples of leaves are shown in **Supplementary Table 2**. The raw reads of leaves with different treatment days all reached more than 45 million, and the matches all reached more than 93%, with excellent similarity to the reference genome. The amount of valid data after removing adapters and filtering all reached more than 95%. The GC content values in each group were above 43%, the Q20 percentage was above 98%, and the Q30 percentage was above 95%. The analysis results indicated that the sequencing was of high quality and reliable data, which could be used for further experimental analysis.

### 3.3 RNA-seq and identification of DEGs during disease progression

**Figure 2:**
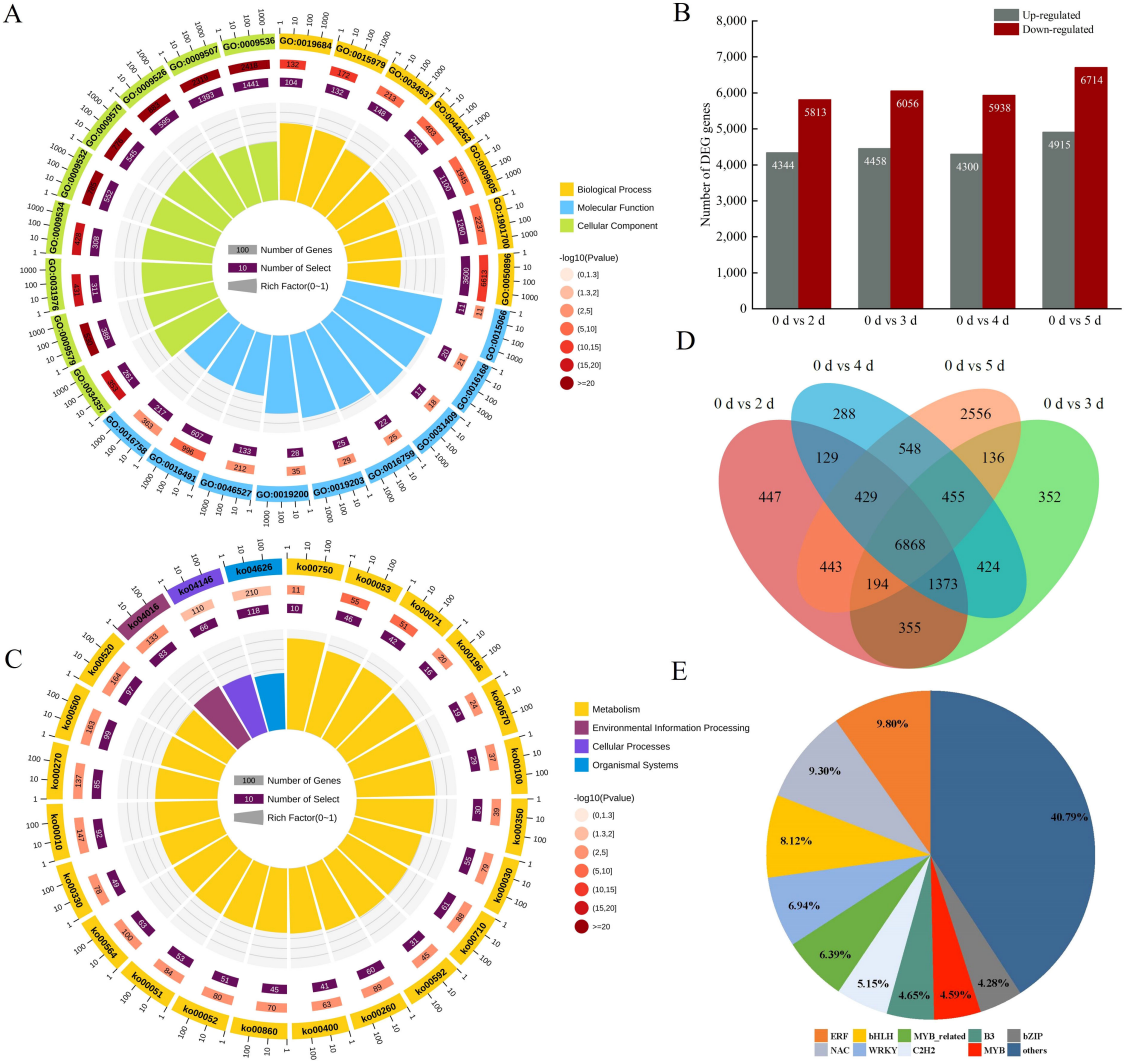
DEGs between samples and functional annotation. **(A)** GO functional enrichment analysis of DEGs (twenty-five significantly enriched GO terms are shown). **(B)** Numbers of DEGs between two samples. DEGs are shown in gray (up-regulated) and red (down-regulated). **(C)** KEGG pathway enrichment analysis of DEGs. (twenty-four significantly enriched GO terms are shown). **(D)** Venn diagram of the DEGs in poplar leaves after inoculation with *A. alternata*. **(E)** Identification of differentially expressed transcription factors.

To study DEGs in poplar during pathogen infestation, gene expression was compared between pathogen-infested and control samples. The number of DEGs gradually increased with the duration of pathogen infestation. Among all the comparisons, the most DEGs were found when comparing the 0 d and 5 d libraries, with a total of 11,629, 4,915 up-regulated, and 6,714 down-regulated (**Figure 2B**). The Venn diagram indicates that unique and shared DEGs exist across groups (**Figure 2D**). The above results suggest that there are more genes involved in the defense response during pathogen infection, and the genes involved in the defense response may be different during different infections. PCA clustering was conducted on the transcriptome data in relation to inoculation status and time points. The results showed that within the PCA space, the CK was entirely distinct from the experimental groups. In contrast, the experimental groups exhibited overlap among themselves in the PCA space (**Supplementary Figure S3**).

### 3.4 GO enrichment and KEGG enrichment analysis of DEGs

The functional classes of DEGs induced by pathogenic infection were obtained by GO enrichment and KEGG enrichment analysis. In the GO enrichment analysis (**Figure 2A**; **Supplementary Table S3**), the most important cellular components (CC) were mainly associated with “plastid stroma” and “chloroplast”. In addition, the most important molecular function (MF) enrichment was related to “oxidoreductase activity” and “chlorophyll binding”, while the bioprocess (BP) was related to “response to abiotic stimulus” and “cellular carbohydrate metabolism processes”. The KEGG database was used to identify pathways showing significant changes (**Figure 2C**; **Supplementary Table S4**), of which “peroxisome”, “MAPK signaling pathway – plant”, “ascorbate and aldehyde metabolism”, “plant-pathogen interactions”, and “amino acid metabolism” were representative. It shows that phytohormones and oxidative enzymes play important roles in poplar in resisting pathogen infection, while the “MAPK signaling pathway” suggests that the expression of genes in poplar is regulated by various signaling substances during pathogen infection. In addition, several TFs were identified among these DEGs (**Figure 2E**), including the families such as bHLH, NAC, ERF, MYB, WRKY, and bZIP (**Supplementary Table S5**). Among the extensive array of TFs that have been the subject of prior investigations, certain TFs have been discovered to possess an extraordinarily intimate connection with the plant’s defense mechanisms against biotic stresses. Prominent examples include MYB, WRKY, and ERF. These TFs engage actively in a series of elaborate regulatory pathways, playing a pivotal role in either activating or suppressing particular genes that are essential for warding off pathogen infection. To illustrate, some TFs are capable of directly binding to the promoter regions of defense-associated genes, thereby precisely adjusting their expression levels in reaction to the encroachment of pathogens. This result suggests that TFs also play an important role in the disease resistance of *Populus simonii × P. nigra*.

### 3.5 Regulation and expression analysis of DEGs related to disease resistance

**Figure 3:**
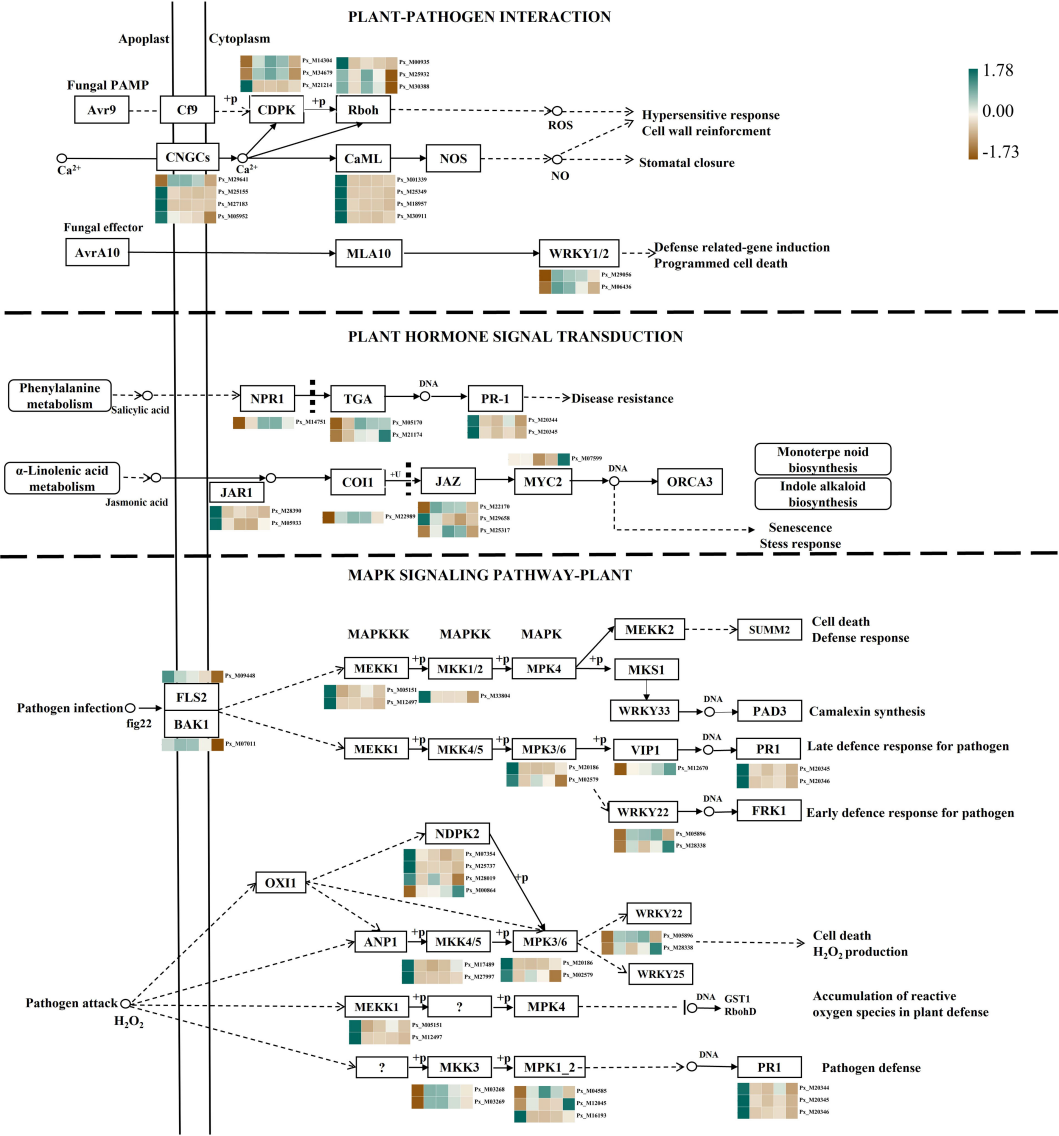
Expression of genes in the metabolic pathway. CDPK, Calmodulin-domain Protein Kinases; Rboh, Respiratory burst oxidase homologue; CNGCs, Cyclic nucleotide-gated channels; CaML, Calcium-mediated signal regulator of cyclophilin ligand gene; WRKY1/2, Transcription factor WRKY1/2; NPR1, Nonexpressor of pathogenesis-related genes 1; TGA, TransGlutaminase Antibody; PR-1, pathogenesis-related protein 1; JAR1, Jasmonate-amido acid synthetase 1; COI1, Coronatine-insensitive protein 1; JAZ, Jasmonate ZIM domain-containing protein; MYC2, Transcription factor MYC2; FLS2, Flagellin-sensitive 2; BAK1, brassinosteroid insensitive 1-associated receptor kinase 1; MEKK1, Mitogen-activated protein kinase kinase 1; MKK1/2, Mitogen-activated protein kinase kinase 1/2; MKK4/5, Mitogen-activated protein kinase kinase 4/5; MPK3/6, Mitogen-activated protein kinase 3/6; VIP1, Vegetable insecticidal protein 1; PR1, Pathogenesis-related protein 1; WRKY22, Transcription factor WRKY22; NDPK2, ucleoside-diphosphate kinase; MKK3, Mitogen-activated protein kinase kinase 3; MPK1_2, Mitogen-activated protein kinase 1_2.

In this research, DEGs associated with plant-pathogen interaction (ko04626), plant hormone signal transduction (ko04075), and the mitogen-activated MAPK signaling pathway (ko04010) exhibited remarkable alterations during pathogen invasion. Pathway figures were then constructed in accordance with the KEGG Pathway database and the expression profiles of these DEGs. The results are shown in **Figure 3** and **Supplementary Table S6**. In the plant pathogen interaction pathway, it can be seen that both PTI and ETI modes are involved in gene expression. The PAMP of fungi penetrates the cell wall and cell membrane, and the CNGCS channel proteins participate in the reaction. After the microbial protein factors are recognized by the transmembrane protein receptors in the plant cell wall or cell membrane, calcium-dependent protein kinases (CDPK) are activated, and then their expression is down-regulated. Down-regulated of the Rboh gene by calcium ions and protein phosphorylation affects the production of ROS, which further results in cell wall strengthening, a superoxide response, and other effects. After AvrA10 in the fungal effector crosses the cell wall and cell membrane, WRKY1-relate and WRKY2-related genes are rapidly expressed, leading to further expression of defense-related genes to induce programmed cell death.

Plant hormones can also participate in the process of plant resistance to pathogen infection. Genes related to JA synthesis, including the amino acid synthase gene JAR1, were significantly down-regulated at all time points. The JA receptor COI1 was up-regulated in the early stage after pathogen inoculation. The signal regulatory factor JAZ, as a negative regulatory factor in JA signaling, showed a trend of first up-regulation and then down-regulation during the post-infection period. The expression level of the MYC2 transcription factor reached its highest on the 5th day of inoculation. Simultaneously involved in the synthesis of salicylic acid, the salicylic acid receptor NPR1 was a star molecule in the plant immune signaling regulatory network, which controls over 2,000 genes related to plant immunity. Its expression reaches its peak on the 4th day.

The plant MAPK cascade pathway has been widely proven to play an important role in biotic stress and plant development, especially in the early signal transduction process after plant injury, which is often composed of three parts: MAPKKK, MAPKK, and MAPK. When the pathogen infected *Populus simonii × P. nigra*, the plant receptor kinase FLS2 began to downregulate expression. The co-receptor BAK1 had the highest expression level on the 3rd day and jointly activated the downstream immune response of the plant. In the cascade reaction, the first two genes of the MEKK class were downregulated on the 2nd day of pathogen inoculation, and then the continuous downregulation of MPK3/6 expression activated the WRKY22 transcription factor and VIP1 promoter through phosphorylation. The WRKY22 transcription factor reached its highest expression level on the 4th day and 5th day of pathogen infection, while the gene in the VIP1 promoter continued to be upregulated during the pathogen infection period, reaching its highest level on the 5th day of inoculation, further activating PR1 for defense response against the late stage of pathogen infection. When pathogens further attacked plants, the level of reactive oxygen species increased in the plant. WRKY22 and WRKY25 promoted plant cell apoptosis and accumulation of reactive oxygen species, while MPK1_2 showed different expression patterns. After binding to DNA, MPK1_2 also activated PR1, thereby completing the plant’s defense response against pathogens.

### 3.6 qRT-PCR validation of RNA-seq results

**Figure 4:**
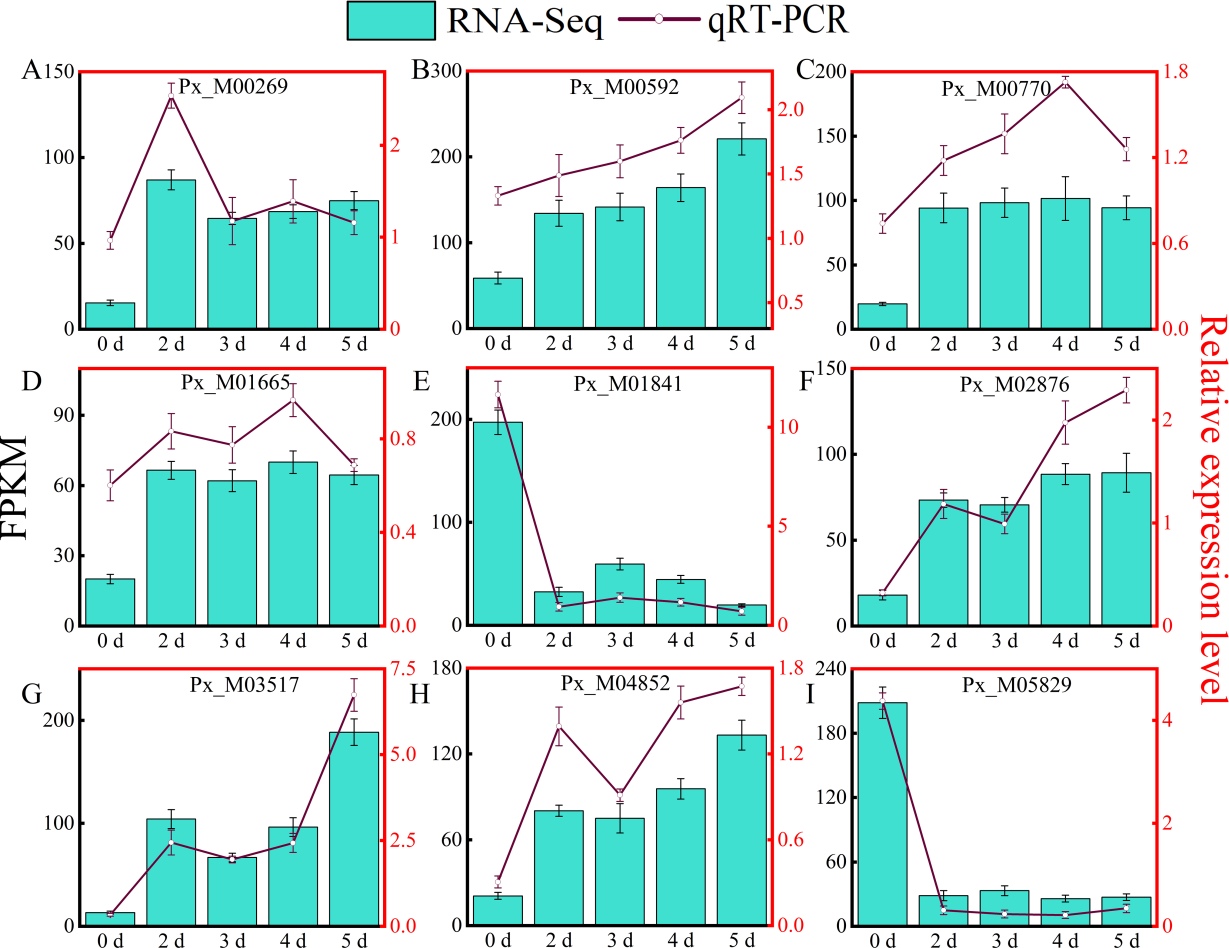
Validation of RNA-Seq Data using qRT-PCR. The part labels: RT-qPCR verification of expression level of nine DEGs related to *A. alternata* identified by RNA sequencing in different time points. The Y-axis on the right indicates the relative gene expression levels analyzed by qRT-PCR, while the Y-axis on the left represents the FPKM value obtained by RNA-seq. The X-axis represents the samples under different treatments time points. Error bars indicate mean ± SE (n = 3) from three independent trials.

To validate the accuracy and repeatability of the RNA-seq data, nine DEGs were randomly selected for qRT-PCR analysis. The RNA-Seq results and qRT-PCR results of the differentially expressed genes of *Populus simonii × P. nigra* at different time points are shown in **Figure 4**. Although the fold of differential gene expression in the qRT-PCR results was not exactly the same as that of the RNA-Seq data, linear regression analysis (**Supplementary Figure S4**) shows that the two trends are similar (R^2^ = 0.8069, *P* < 0.001), these results verified the accuracy and reliability of the RNA-Seq data.

### 3.7 WGCNA analysis identifies key genes for disease resistance in *Populus simonii* × *P. nigra*

**Figure 5:**
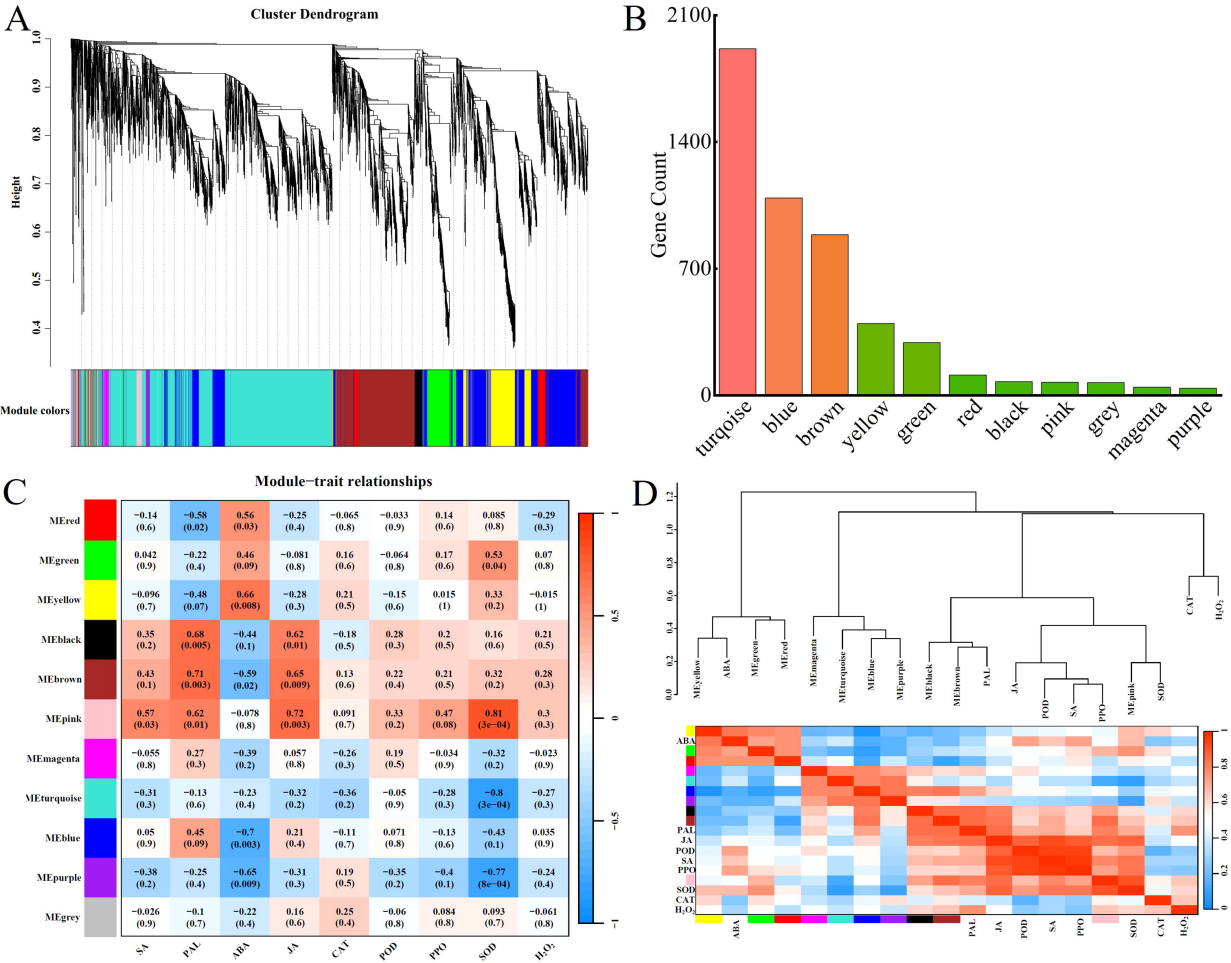
Weighted gene co-expression network analysis of DEGs. **(A)** Cluster dendrogram of all DEGs. **(B)** Distribution of gene counts in modules. **(C)** Module-trait relationship heatmap of 11 modules. **(D)** Heatmap of inter-module correlations.

Constructing a clustering tree based on gene expression, we merge genes with similar expression patterns into the same branch. Then, based on the similarity of module feature values, we merge modules with similar expression patterns to obtain partitioned modules, which are represented by different colors (**Figure 5A**). Using WGCNA analysis, we performed correlation analysis and clustering based on the FPKM values of DEGs. The genes with higher correlation were assigned to the same module, and different colors in the figure represented different modules. Eventually, all the DEGs were classified into a total of 11 modules. The number of genes in each module is shown in **Figure 5B**. Moreover both up-regulated and down-regulated genes were present in each module, and the heat map gene co-expression networks are shown in **Figure 5C**. Based on the correlation analysis of the trend of gene expression modules with disease resistance traits, Pearson’s correlation coefficient (|R| > 0.6) and the magnitude of the significant P-value (*P* < 0.05) were used as the screening criteria, and it was found that the yellow module, the black module, the brown module, and the pink module were significantly and positively correlated with physiological and biochemical indicators. Among them, the yellow module was positively correlated with ABA, the black module was positively correlated with PAL and JA, the brown module was positively correlated with PAL and JA, and the pink module was positively correlated with PAL, JA, and SOD. Physiological and biochemical indicators are also clustered together (**Figure 5D**). The results indicate that the characterized genes of these four modules are likely to be associated with resistance to *Populus simonii × P. nigra* against *A. alternata*.

### 3.8 Enrichment analysis of disease resistance-related module genes

**Figure 6:**
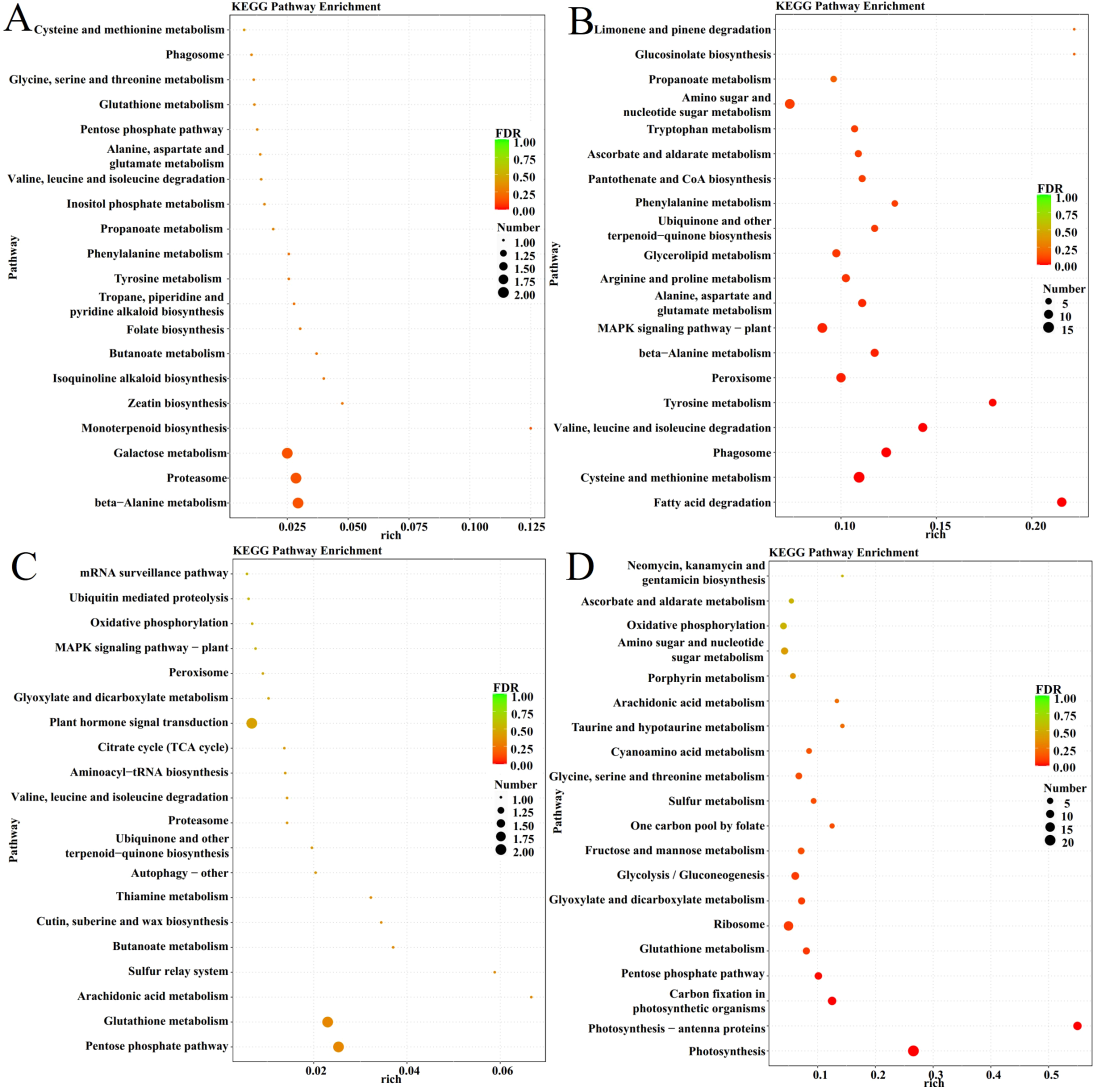
KEGG Pathway Enrichment analysis of different Modules. **(A)** Black module. **(B)** Brown module. **(C)** Pink module. **(D)** Yellow module.

The DEGs in these several disease resistance-related modules were further analyzed using KEGG enrichment to understand the expression of their related genes, and the results are shown in **Figure 6** and **Supplementary Table S7**. The black module was enriched with pathways that mainly focus on galactose metabolism, proteasomes, and alanine metabolism; the brown module, whose enriched pathways mainly focus on the MAPK signaling pathway, phenylpropane biosynthesis, phenylalanine, and proline metabolism; the pink module, whose enriched pathways mainly focus on the phytohormone signaling pathway, glutathione metabolism, and pentose phosphate pathway; and the yellow module, whose enriched pathways mainly focus on photosynthesis and carbon metabolism.

### 3.9 Screening and functional annotation of Hub genes

**Figure 7:**
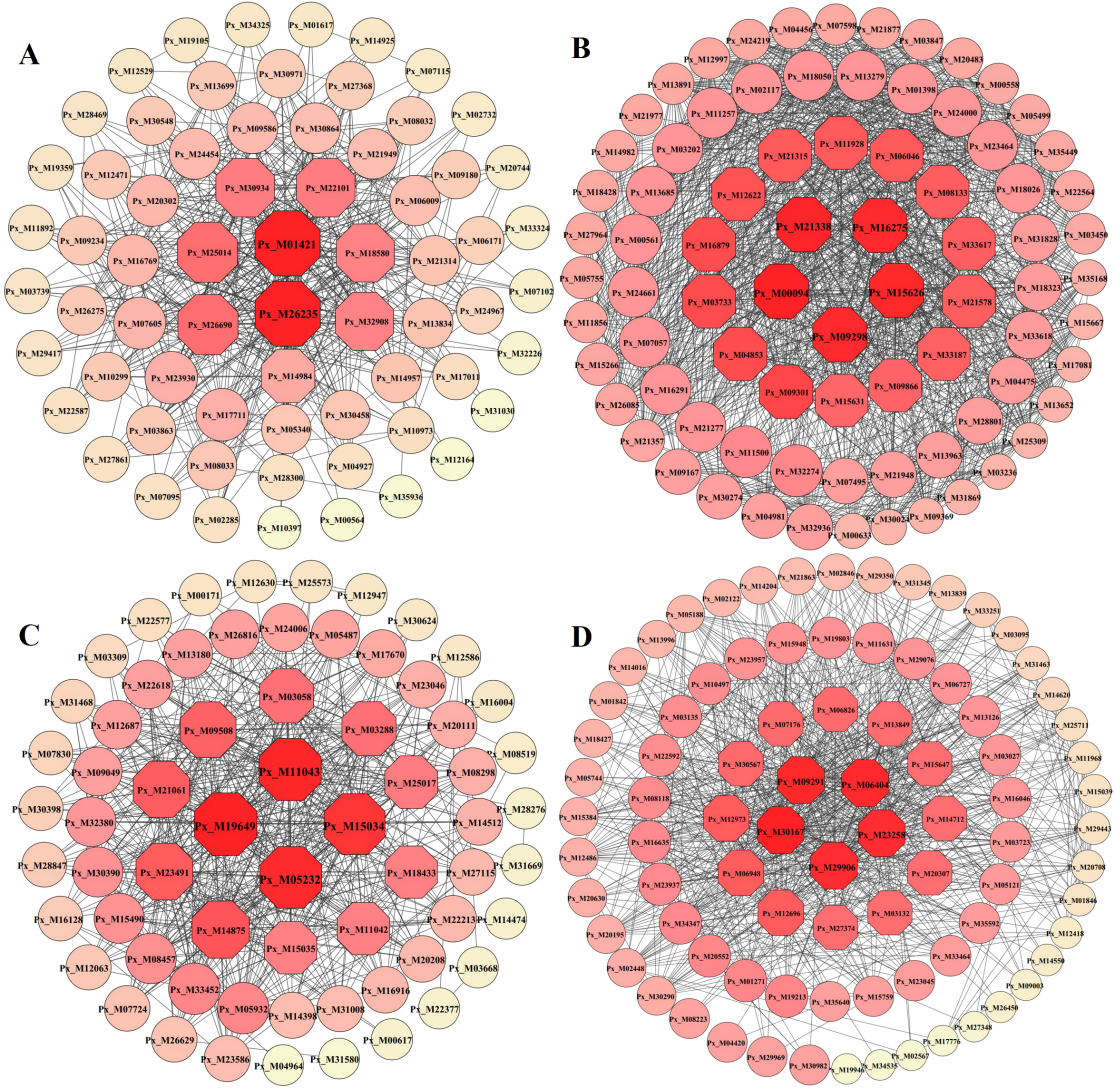
Interaction networks of Hub genes in the module. Node size and color depth represent node connectivity. **(A)** Black module. **(B)** Brown module. **(C)** Pink module. **(D)** Yellow module. The hub genes identified as highly interconnected nodes based on degree value, and the depth of the colour represented the degree value of nodes (the darker the colour, the greater the degree).

Hub genes are genes that are in an important position in the co-expression network, indicating a high degree of association with other genes. Based on the degree, select the genes with high connectivity ranking within the yellow, black, brown, and pink modules in sequence, and then utilize Cytoscape to construct an interoperability network diagram to further analyze the core genes in the network diagram. The results showed (**Figure 7A–D**; **Supplementary Table S8**) that a total of 16 hub genes were screened in the four modules; multiple genes were located at the core of their respective networks.

Using the comparison tool on the InterProscan website, the protein sequences of the core genes were compared with all the protein sequences of Populus, and the ones with the highest comparison results were selected for their functional annotation. The functional annotation information of the core genes is shown in **Table 1**. The black module core genes were annotated as GLK1/2 transcriptional activators; the brown module core genes were annotated as a variety of enzymes and proteins related to photosynthetic respiration; the pink module core genes were annotated as glucose-6-phosphate dehydrogenase and 14-3-3 proteins; and the yellow module core genes were annotated as chlorophyll A-B binding proteins and CbbY proteins, etc.

**Table 1:** Module hub gene function annotation.

Mudule	Gene ID	Gene annoation
Meblack	Px_M26235	Hypothetical protein
	Px_M01421	Transcription activator GLK1/2-like
Mebrown	Px_M21338	Acyl-CoA dehydrogenase
	Px_M15626	Aspartate/other aminotransferase
	Px_M00094	DNA Cytosine-5 Methyltransferase
	Px_M09298	Hypothetical protein
	Px_M16275	Sugar/inositol transporter
Mepink	Px_M15034	Hypothetical protein
	Px_M19649	Hypothetical protein
	Px_M11043	Glucose-6-phosphate dehydrogenase
	Px_M05232	14-3-3 protein
Meyellow	Px_M29906	Chlorophyll A-B binding protein
	Px_M23258	Chlorophyll A-B binding protein
	Px_M06404	Glyceraldehyde-3-phosphate dehydrogenase, type I
	Px_M09291	Triose phosphate/phosphoenolpyruvate translocator
	Px_M30167	Protein CbbY-like

**Figure 8:**
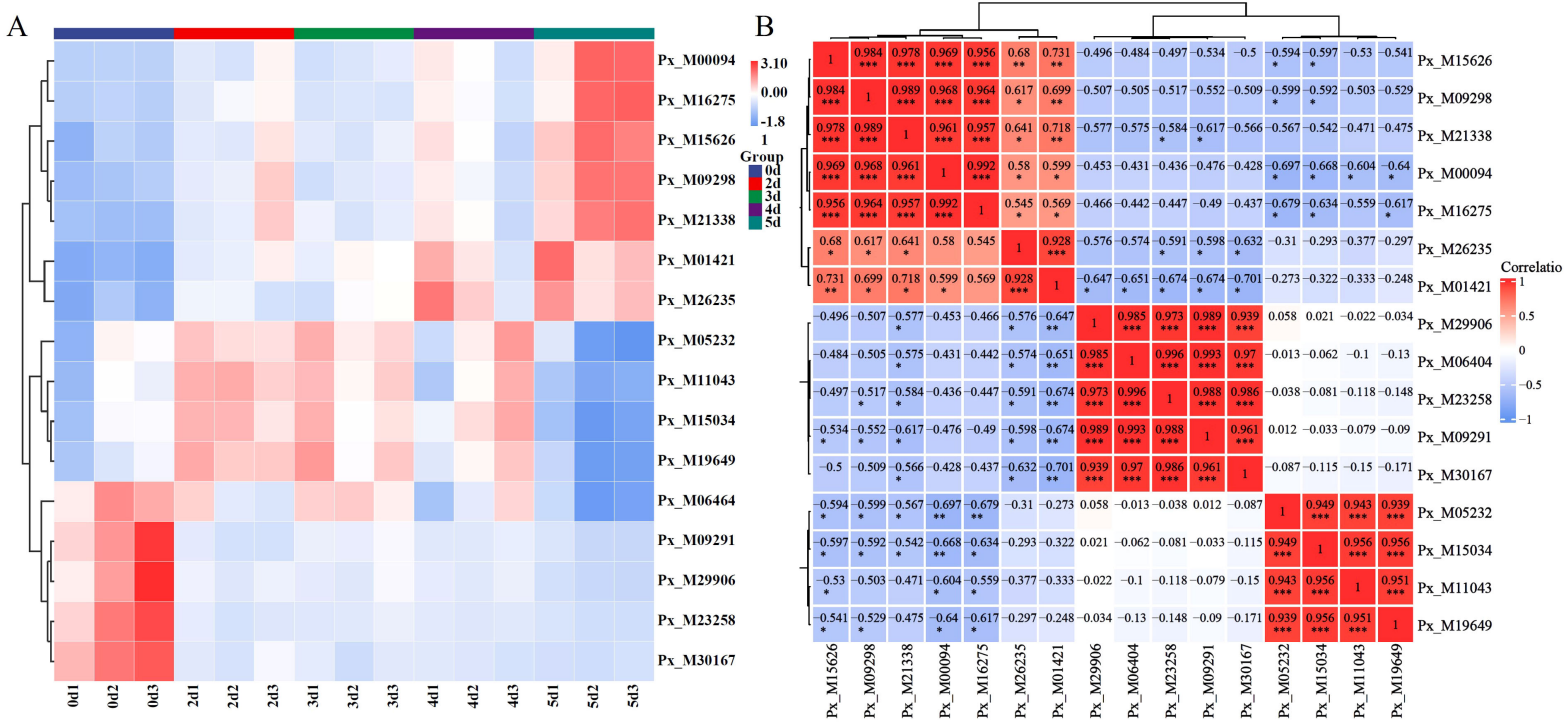
Expression of hub genes. **(A)** Cluster heatmap of Hub genes. **(B)** Pearson correlation of hub genes. (* P < 0.05, ** P < 0.01, *** P < 0.001).

The expression of Hub genes in the regulatory network is shown in **Figure 8A**. Different genes exhibited different expression patterns. Genes such as Px_M00094 were significantly upregulated when infected, while genes such as Px_M05232 showed a trend of upregulation followed by downregulation. On the 2 d of infection, genes such as Px_M09291 were significantly downregulated, and their expression levels remained relatively stable thereafter. By Pearson correlation matrix analysis (**Figure 8B**), we identified hub gene pairs with significant correlations in expression levels, with most genes showing a significant positive correlation with others.

## 4 Discussion

### 4.1 Physiological and biochemical indices in response to pathogen infestation

Phytohormones such as SA, JA, ABA, and enzymes such as POD, PPO, SOD, CAT, and PAL play crucial roles throughout the life cycle of the plant, especially in the response to a variety of biotic and abiotic stresses. When plants are infested by external pathogens, the cells break the existing steady state, and the level of ROS in the cells increases. The antioxidant enzyme system of plants contains SOD, CAT, and PAL (Imahori et al., 2008), and the balance between the activities of a variety of antioxidant enzymes is a key factor in determining the homeostasis of O_2_. and H_2_O_2_. SOD acts as the first line of defense among the defense enzymes, converting O_2_. to H_2_O_2_, while CAT scavenges excess H_2_O_2_ (Mittler, 2002). After inoculation with leaf blight pathogens, the activity of SOD increased significantly on 2 d and 3 d and decreased on 4 d, whereas the trend of CAT was more or less the same as that of SOD, while the content of H_2_O_2_ began to increase on 2 d, due to the production of large amounts of H_2_O_2_ in the course of SOD action. The activities of PAL, POD, and PPO also showed a tendency to increase and then decrease with further infestation of the pathogen and its growth and spread within the leaves. Populus infected by *Marssonina brunnea* also showed a significant increase in SOD and CAT activities (Chen et al., 2020a). Tomatoes inoculated with seaweed extracts showed a significant increase in total free amino acid content and antioxidant enzyme activities (CAT, POD) in the aboveground and root systems, and the plant resistance to *Fusarium oxysporum* was improved (El-Sheekh et al., 2020). PPO is involved in the process of oxidation of phenolics to quinones in plants, which increases their toxicity to pathogens and thus improves disease resistance. Several studies have shown that the activity of PPO shows a positive correlation with the disease resistance of plants (Khodadadi et al., 2020; Attia et al., 2022). PAL, as a key enzyme in the metabolism of phenylpropanes, can promote the production of secondary metabolites such as phenolics, flavonoids, and lignin, which are closely associated with plant disease resistance (Saberi Riseh et al., 2022; Zhu et al., 2024).

Further studies on the changes in plant endogenous hormones showed that SA, as an important plant endogenous hormone for plant resistance to pathogens, can enhance plant disease resistance through three pathways, namely, regulating enzyme activity, inducing the production of reactive oxygen species, and stimulating the expression of relevant disease process proteins. After inoculation with *A. alternata*, the SA content did not change significantly on 2 d, but increased rapidly on 3 d, then began to decline. The same study showed an increase and then a decrease in SA accumulation in wheat after inoculation with rust fungi, while the rust resistance gene *Yr10* was highly expressed (Gao et al., 2021). When Populus are infected with rust disease, the content of JA decreases in tolerant individuals, while it rises in intolerant ones. In Populus that lack tolerance to rust infection, the content of SA often shows an upward trend (Chen et al., 2021). JA inhibits the growth and development of pathogens by inducing plants to produce alkaloids and phenolic acids that bind to the proteins of pathogens, thereby poisoning them. At the same time, JA also regulates the induced systemic resistance of plants for defense responses when the plant body is infested with pathogens (Cox, 2022). ABA, as a stress phytohormone, regulates plant adaptation to stressful environments. When pathogens infest leaves, leaves increase the permeability of chloroplast membranes to abscisic acid, which triggers the synthesis system to synthesize abscisic acid (Lin et al., 2021). Excessive abscisic acid synthesis can lead to leaf senescence (Chen et al., 2020b). In this study, the abscisic acid content in the leaves of *Populus simonii × P. nigra* continued to increase after inoculation with *A. alternata*, which improved leaf resistance but also led to premature leaf abscission. Alterations in the activity levels of redox - associated enzymes and protective enzymes, as well as fluctuations in hormone concentrations, all play a pivotal part in stress response mechanisms.

### 4.2 Response of disease resistance-related metabolic pathways to pathogen infestation

In the metabolic pathway of plant pathogen interactions, plant cell surface pattern recognition receptors (PRRs) and intracellular protein kinases are activated. Concurrently, CNGCS channel proteins and CDPK are induced for expression, which further generates ROS and activates the plant defense response (Ngou et al., 2021). In the present study, when inoculated with pathogenic bacteria, both PTI and ETI modes were engaged, leading to the expression of the CNGCS channel protein gene, changes in Ca^2+^ concentration, and the activation of genes such as CDPK, Rboh, and CaM/CML. These responses contribute to strengthening the leaf cell wall and generating a superoxide response to combat pathogenic bacteria. Similarly, the research demonstrated that elevated cytoplasmic calcium is essential for PAMP-triggered PTI, and the calcium channel protein OsCNgC9 in rice can positively regulate rice resistance to rice blast (Pruitt et al., 2021).

Among the phytohormone signaling pathways, the JA signaling pathway is involved in plant defense against pathogens or inducing induced systemic resistance (ISR) in plants through two branches, MYC and ERF. In this study, the expression of the MYC2 transcription factor was consistently up-regulated and peaked at 5 days post-inoculation. It has been shown that resistance to leaf blight in rice can be enhanced by overexpressing the MYC2 gene in rice during JA-mediated plant immunization, whereas genes such as ERF3 in wheat and ERFs in rice are involved in the JA-mediated defense response (Zhai et al., 2013). Overexpressed wheat transgenic lines show enhanced resistance to powdery mildew. Genes related to SA-mediated defense, such as those in the PR family and PAD family, were significantly up-regulated in the overexpressed plants, along with a significant increase in resistance to leaf rust in wheat (Kumar et al., 2014). NPR1, TGA, and PR1 were induced to be up-regulated and expressed in this study, which was consistent with the results of previous studies. Huang conducted measurements on the RNA Seq of *P. davidiana × P. bolena* in response to *A. alternata* infection. It was discovered that the DEGs were predominantly concentrated in the “plant hormone signal transduction” pathway. Moreover, the DEGs associated with JA biosynthesis and JA signal transduction were continuously activated (Huang et al, 2022). When *P. tremuloides* is infected by *Sphaerina* spp., the JA, SA, and ABA signaling pathways are also involved and exhibit high levels of expression (Foster et al, 2015).

The plant MAPK cascade pathway is closely related to plant adversity signaling. It has been shown that the MAPK signaling pathway is activated by ROS, a signaling molecule that acts as an activator of the stress response pathway (Jalmi and Sinha, 2015). Moreover, MEKK1, MKK4/5, and MPK3/6 in the signaling pathway are similarly activated in response to pathogen stress. In this study, after the activation of the receptor kinase FLS2 and the co-receptor BAK1, the expression of related genes in MEKK1, MKK4/5, and MPK3/6 was induced. Meanwhile, MAPK was also involved in the biosynthesis of JA, SA, and ET, as well as their signaling pathways (Zhou et al., 2022). It triggered the activation of inducible transcription factors to activate the expression of downstream resistance genes (Li et al., 2022), thereby improving the resistance of plants to biotic and abiotic stresses. The KEGG pathway associated with disease resistance is truly an exceedingly intricate and elaborate regulatory mechanism, which occupies a central and indispensable position in safeguarding plants against the encroachment of diseases.

### 4.3 Functional analysis of Hub genes in the regulatory network of disease resistance-related genes

WGCNA is an effective method for analyzing the gene expression status across multiple samples. Genes with similar expression patterns in physiological processes or different phases can be recognized as functionally similar and subsequently clustered into a module (Duan et al., 2023). Hub genes can then be predicted by analyzing the correlation between the genes within the module and the phenotype.

In this study, we constructed a co-expression network of genes directly related to poplar leaf blight by screening the weights among the characterized genes in multiple modules. A total of 16 Hub genes were screened, and some of these Hub genes have been reported to be associated with biotic stresses in other plants. Among them, GLK (Golden2-like), as a plant-specific transcription factor, is heavily involved in regulating the development of chloroplasts throughout all stages of plant growth and development (Tu et al., 2022). Chloroplasts play a key role in plant immunity by producing a variety of defense signaling molecules. The GLK1/2 transcriptional activators negatively regulate the process of plant response to stress and activate the expression of downstream genes, such as WRKY40, which in turn activates the response pathway to ABA (Ahmad et al., 2019). Mutants of GLKs showed greater tolerance to osmotic and salt stress compared to wild-type plants. Meanwhile, GLK1/2 can regulate chlorophyll biosynthesis and flavonoid accumulation in response to specific light signals, which further affects the properties of tea leaves (Liu et al., 2023). Flavonoids play an important role in response to both biotic and abiotic stresses. In addition, GLK genes are also involved in regulating plant defense responses. For example, *GLK1* overexpression increased resistance to the pathogenic *Fusarium graminearum* (Savitch et al., 2007), while *Arabidopsis thaliana* ectopically expressing the peanut *AhGLK1b* gene also showed strong resistance to the bacteria *PstDC3000* and *Ralstonia solanacearum* (Ali et al., 2020). In this study, GLK was found to be upregulated as well, which is in line with the findings of previous research.

The 14-3-3 protein family is involved in the regulation of plant immunity to pathogens and plays an important role in plant-pathogen interactions. It has been shown that *Nicotiana benthamiana* 14-3-3h interacts with translationally controlled tumor protein (TCTP), a susceptibility factor of potato Y virus (PVY). Furthermore, the antiviral activity of three Nb14-3-3h dimerization-deficient mutants was significantly reduced, suggesting that Nb14-3-3h dimerization is essential for the inhibition of PVY infection (Fang et al., 2024). 14-3-3 proteins can respond to different stresses by altering their expression levels or properties. Gene expression analysis showed that *ZmGF14-6*, encoding a maize 14-3-3 protein, was up-regulated under fungal infestation and salt stress and down-regulated under drought stress (Campo et al., 2012). Additionally, our research findings suggested that the onset of fungal infection induces the upregulation of 14-3-3 expression. In *Arabidopsis*, the 14-3-3 protein responds to pathogens by interacting with a variety of defense-associated proteins in plants, and overexpression of 14-3-3 leads to a hypersensitive response and enhances resistance to powdery mildew through a SA-dependent signaling pathway (Yang et al., 2009).

Cytosine-5 methyltransferase also plays an important role in DNA methylation and epigenetics, and DNA methylation is very sensitive to the effects of environmental stresses. We found that the Cytosine-5 methyltransferase gene was persistently upregulated as a response to pathogen invasion. The rice variety Wase Aikoku 3 is not resistant to the wilt disease *Xanthomonas oryzae* when it is at the seedling stage, whereas it exhibits resistance at the adult plant stage. Artificial inoculation of rice at both seedling and adult stages revealed, using the MSAP method, a higher number of highly methylated polymorphic sites in adult-stage rice than in seedlings (Sha et al., 2005). These polymorphic methylated sites are likely to influence rice resistance. Akimoto et al. induced wilt resistance in a rice line-2 strain using the demethylation reagent 5-azodeoxycytosine. They screened line-2 and wild-type rice for polymorphic fragments using the MSAP technique and obtained an *Xa21G* clone, which was found to be very similar to the wilt-resistant rice gene. The promoter of *Xa21G* in line-2 was unmethylated, while it was fully methylated in the wild type, leading to high expression of *Xa21G* in line-2 (Akimoto et al., 2007). Therefore, it is possible to increase the expression of this gene by reducing the methylation level of the *Xa21G* promoter, thereby achieving wilt resistance. These hub genes, which play a pivotal role in the co-expression network, are crucial for the defense which may indicate a potential role in defense process of *Populus simonii × P. nigra* against *A. alternata.*

## 5 Conclusion

In this study, we correlated physiological and biochemical indicators, such as phytohormone and enzyme activities, with gene expression profiles using WGCNA co-expression network analysis to investigate the molecular mechanism of resistance to *A. alternata* in *Populus simonii × P. nigra*. Upon being infected by the pathogen, the physiological and biochemical indicators of Populus simonii × P. nigra change significantly as a means to resist its invasion. The study showed that several transcription factors related to growth, development, and disease resistance, such as ERF, MYB, bZIP, and WRKY, exhibited differential expression. Through WGCNA, we identified gene modules significantly associated with indicators such as ABA, PAL, JA, and SOD, and the genes in these modules are involved in key biological stress, signal transduction, cell wall, and photosynthesis-related biological processes. In addition, Hub genes in the co-expression network include the GLK1/2 transcriptional activator, 14-3-3 protein, and cytosine 5 methyltransferases, which play important roles in regulating physiological activities and enhancing plant defenses. This study reveals that these highly interconnected hub genes play an important role in the defense of poplar against *A. alternata*, thereby further understanding the molecular mechanisms of plant response to biotic stresses. In the subsequent stage, we will conduct operations such as gene overexpression and gene knockout on the hub genes. These experiments aim to validate their disease resistance functions and to develop Populus varieties with enhanced resistance to *A. alternata*.

## Data Availability

The datasets presented in this study can be found in online repositories. The names of the repository/repositories and accession number(s) can be found in the article/**Supplementary Material.**
