# Supplementary material for: Integrative transcriptome and WGCNA analysis reveal key genes mainly in response to Alternaria alternata in Populus simonii × P. nigra
Source: Front Plant Sci. 2025 Feb 17;16:1540718. doi: 10.3389/fpls.2025.1540718 (PMC11873080; doi:10.3389/fpls.2025.1540718)
Supplement: Supplementary file 1 [file DataSheet1.docx]

Supplementary Material

# Supplementary Figures and Tables

## Supplementary Figures


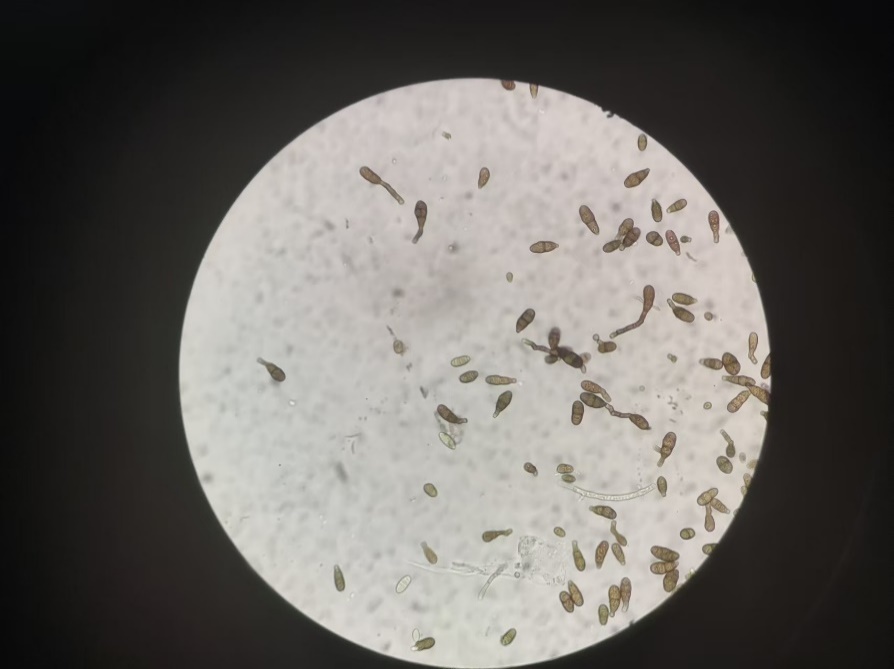


**Supplementary Figure S1.** Spore microscopy image of *Alternaria alternate*.


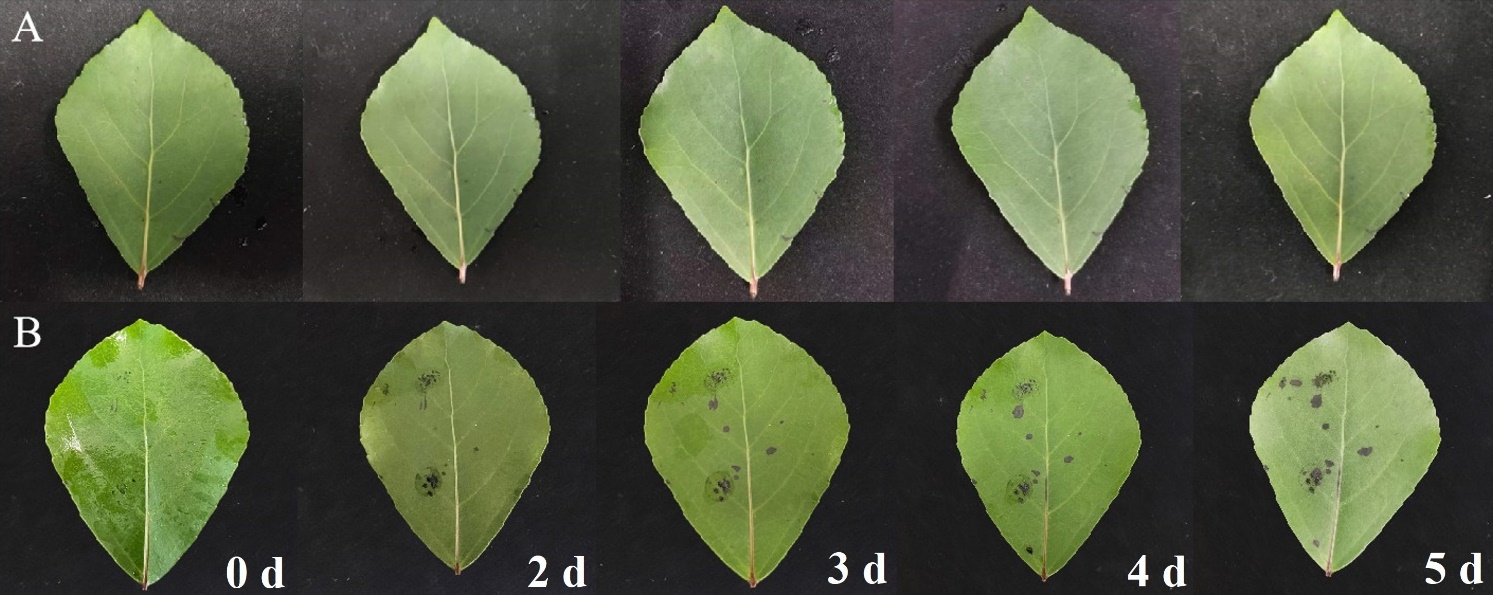


**Supplementary Figure S2.** Phenotypic changes in *Populus.simonii* × *P.nigra*. A: CK. B: pathogen inoculation. 0 d:CK, 2 d: Two days after inoculation with *A. alternate* on leaves, 3 d: Three days after inoculation with *A. alternate* on leaves, 4 d: Four days after inoculation with *A. alternate* on leaves, 5 d: Five days after inoculation with A. alternate on leaves.


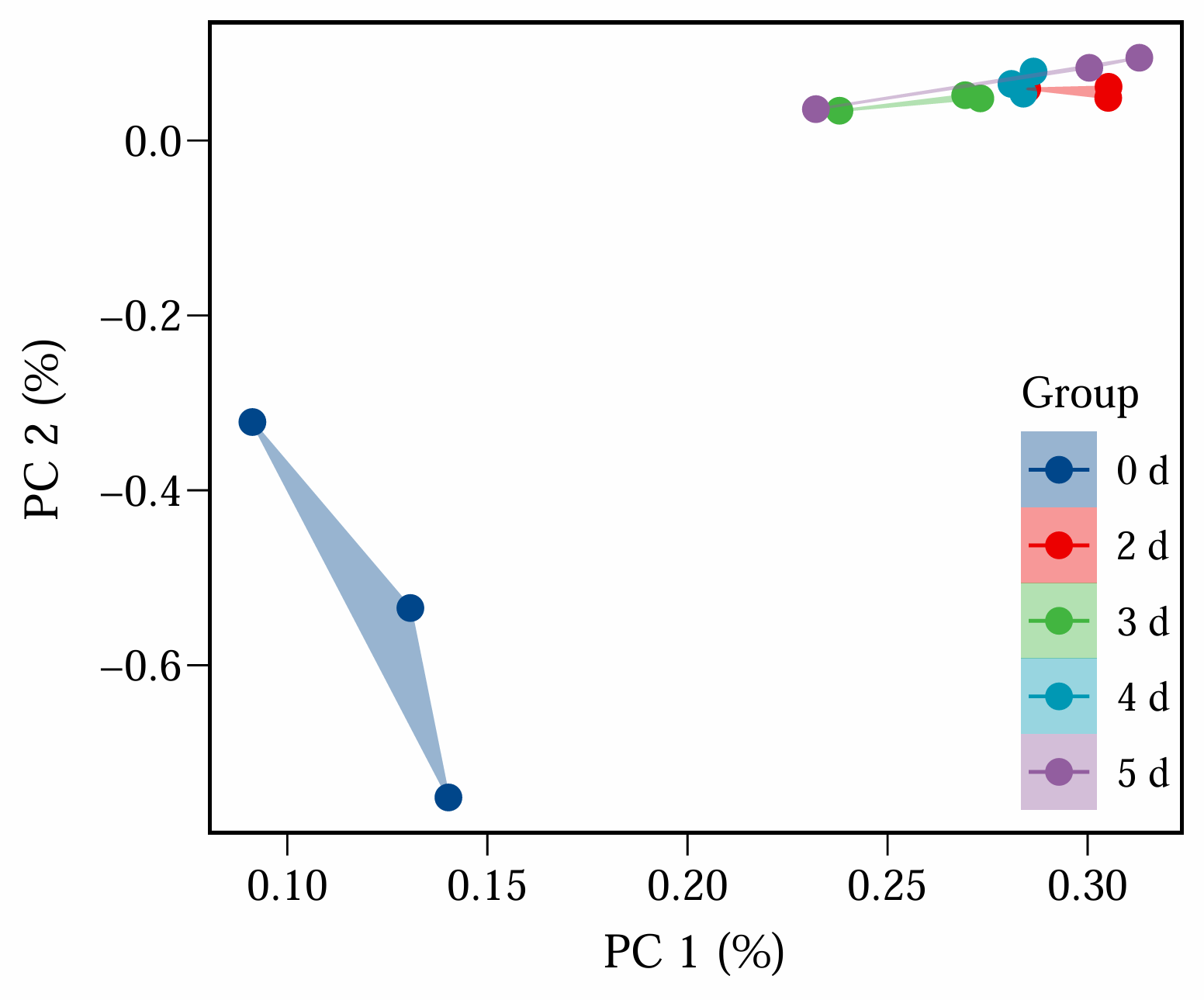


**Supplementary Figure S3.** PCA plot of the RNA samples


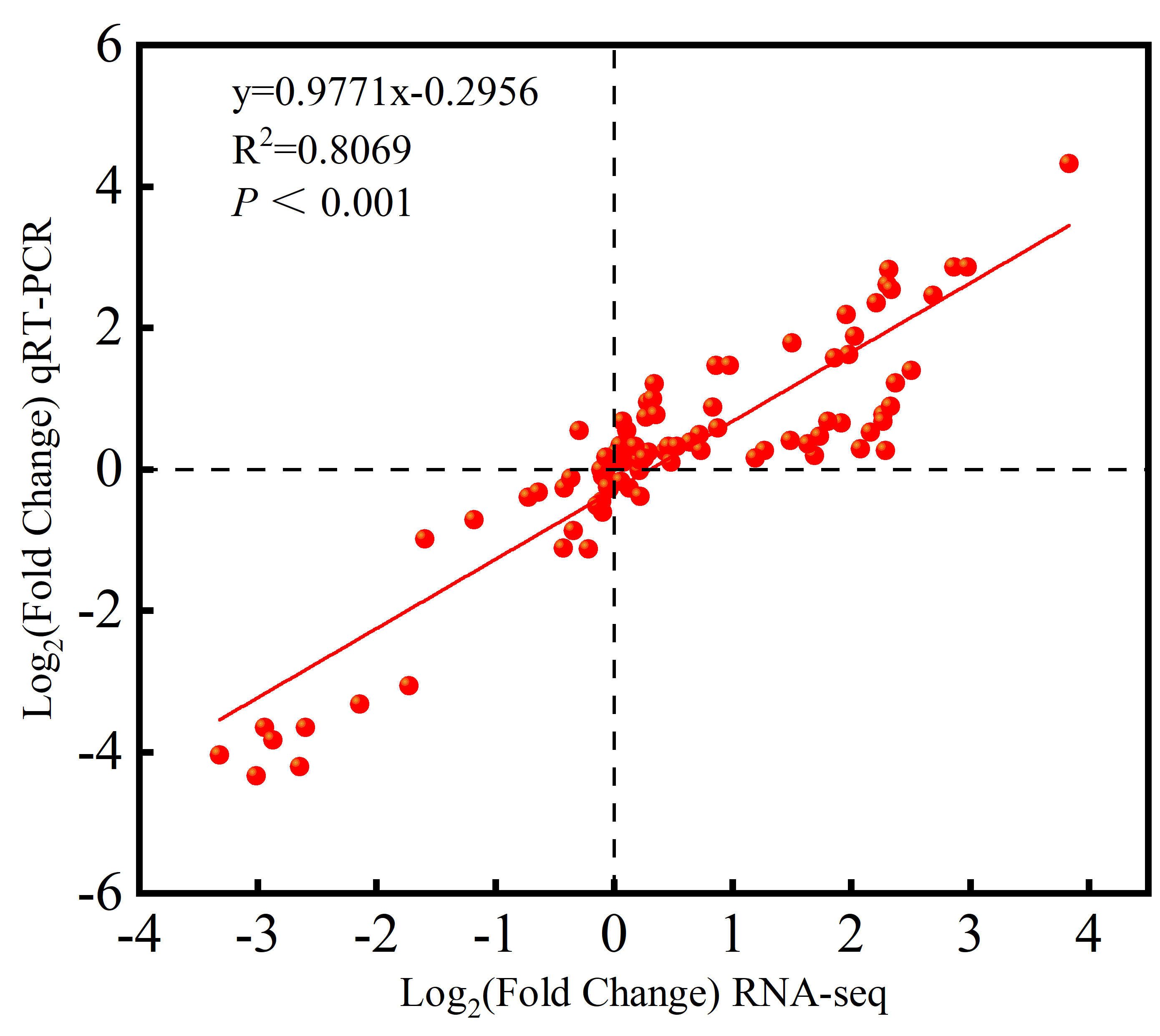


**Supplementary Figure S4.** Linear regression of RNA-Seq and qRT-PCR data that are expressed as a log2 fold change.

## Supplementary Table

**Supplementary Table S1.** Primers for qRT-PCR verification of DEGs.

**Supplementary Table S2.** Reads quality of transcriptome.

**Supplementary Table S3.** GO enrichment of DEGs is induced by pathogenic infection.

**Supplementary Table S4.** KEGG enrichment of DEGs is induced by pathogenic infection.

**Supplementary Table S5.** DEGs information related to TFs.

**Supplementary Table S6.** Detailed information on disease resistance related DEGs.

**Supplementary Table S7.** Detailed information of genes in different disease resistance related modules.

**Supplementary Table S8.** Expression of 16 hub genes.
